# Autologous Tooth Graft: Innovative Biomaterial for Bone Regeneration. Tooth Transformer^®^ and the Role of Microbiota in Regenerative Dentistry. A Systematic Review

**DOI:** 10.3390/jfb14030132

**Published:** 2023-02-27

**Authors:** Angelo Michele Inchingolo, Assunta Patano, Chiara Di Pede, Alessio Danilo Inchingolo, Giulia Palmieri, Elisabetta de Ruvo, Merigrazia Campanelli, Silvio Buongiorno, Vincenzo Carpentiere, Fabio Piras, Vito Settanni, Fabio Viapiano, Denisa Hazballa, Biagio Rapone, Antonio Mancini, Daniela Di Venere, Francesco Inchingolo, Maria Celeste Fatone, Andrea Palermo, Elio Minetti, Felice Lorusso, Antonio Scarano, Salvatore Sauro, Gianluca Martino Tartaglia, Ioana Roxana Bordea, Gianna Dipalma, Giuseppina Malcangi

**Affiliations:** 1 Department of Interdisciplinary Medicine, University of Bari “Aldo Moro”, 70124 Bari, Italy; 2 PTA Trani-ASL BT, Viale Padre Pio, 76125 Trani, Italy; 3 College of Medicine and Dentistry Birmingham, University of Birmingham, Birmingham B4 6BN, UK; 4 Department of Biomedical, Surgical, and Dental Science, Università Degli Studi di Milano, 20122 Milan, Italy; 5 Department of Innovative Technologies in Medicine and Dentistry, University of Chieti-Pescara, 66100 Chieti, Italy; 6 Dental Biomaterials and Minimally Invasive Dentistry, Department of Dentistry, Cardenal Herrera-CEU University, CEU Universities, C/Santiago Ramón y Cajal, s/n., Alfara del Patriarca, 46115 Valencia, Spain; 7 Department of Biomedical, Surgical and Dental Sciences, School of Dentistry, University of Milan, 20100 Milan, Italy; 8 UOC Maxillo-Facial Surgery and Dentistry Fondazione IRCCS Cà Granda, Ospedale Maggiore Policlinico, 20122 Milan, Italy; 9 Department of Oral Rehabilitation, Faculty of Dentistry, Iuliu Hațieganu University of Medicine and Pharmacy, 400012 Cluj-Napoca, Romania

**Keywords:** tooth transformer^®^, biomaterials, autologous graft, tooth graft, demineralized dentine matrix, microbiota, osteogenic material, regenerative dentistry, bone remodeling, morphogenetic proteins

## Abstract

Different biomaterials, from synthetic products to autologous or heterologous grafts, have been suggested for the preservation and regeneration of bone. The aim of this study is to evaluate the effectiveness of autologous tooth as a grafting material and examine the properties of this material and its interactions with bone metabolism. PubMed, Scopus, Cochrane Library, and Web of Science were searched to find articles addressing our topic published from 1 January 2012 up to 22 November 2022, and a total of 1516 studies were identified. Eighteen papers in all were considered in this review for qualitative analysis. Demineralized dentin can be used as a graft material, since it shows high cell compatibility and promotes rapid bone regeneration by striking an ideal balance between bone resorption and production; it also has several benefits, such as quick recovery times, high-quality newly formed bone, low costs, no risk of disease transmission, the ability to be performed as an outpatient procedure, and no donor-related postoperative complications. Demineralization is a crucial step in the tooth treatment process, which includes cleaning, grinding, and demineralization. Since the presence of hydroxyapatite crystals prevents the release of growth factors, demineralization is essential for effective regenerative surgery. Even though the relationship between the bone system and dysbiosis has not yet been fully explored, this study highlights an association between bone and gut microbes. The creation of additional scientific studies to build upon and enhance the findings of this study should be a future objective of scientific research.

## 1. Introduction

Regenerative medicine has received a lot of attention and development in the medical field in recent decades. The study of biomaterials has yielded excellent and predictable results, thanks partly to biotechnology.

The study of bone tissue regeneration, which is widely used in dentistry, has piqued the researchers’ interest [1]. Graft material must function as an osteoconductive scaffold, provide a mineral substrate, contain osteoinductive cells, platelet growth factors, and cell differentiation factors, and be decontaminated [2,3].

Furthermore, the grafts must not only fill and occupy the space of the bone defect, but also maintain the clot and/or provide proteins and/or release cells contained within them. In order to allow the adhesion of osteoblasts and osteoclasts and favor osteoid formation in the first phases of bone regeneration, the granules must have a specific size (500–1000 µm), be manageable and plastic during the surgical phase, as well as have spaces between them that can allow rapid neovascularization [4].

Based on their properties and origins, scaffolds are classified into [5]:

- autologous or allographic human bone, i.e., taken from another donor (4%)
- xenographic: animal bone, coral (54%)
- alloplastic: bioglass, hydroxyapatite (HA), tricalcium β-phosphate (TCP), polyglycolic, and polylactic acid derivatives (33%)
- other (9%)

All studies have confirmed that autologous bone grafting is the gold standard.

This type of graft cannot induce adverse immune reactions because it is autologous, has osteogenic, osteoinductive, and osteoconductive capabilities and stimulates osteoproliferation with rapid healing. However, the need for an additional invasive operation, increased morbidity, pain, and the difficulty of the harvesting areas, reduce the rates of use of autologous bone grafting [6]. Furthermore, growth factors are released only after the graft has been completely resorbed [7,8].

Therefore, grafts of various origins (allographic, xenographic, and alloplastic) have been used for regenerative techniques [9,10]. The majority of these grafts only have osteoconductive properties and very long resorption times (coral and HA) or short resorption times (polyglycolic and polylactic acid derivatives), while others pose risks of immune reactions and infections (allografts), are incompatible with religious cultures, and are expensive on the market, despite all studies showing the good bone regenerative efficacy of these grafts [11,12].

In recent years, special attention has been given to dentine as a biomaterial in bone regeneration [13]. An analysis of its biochemical composition has revealed similarities with bone tissue. Dentine, like bone tissue, is 61% inorganic (HACa_5_(PO_4_)_3_OH, with crystals that are 10 times larger than bone and 300 times smaller than enamel. A total of 90% of the organic component (39%) is collagen: collagen type I (95%), and collagen types III, V, and XII (5%). Collagen gives resilience and elasticity and makes the structure resistant to fractures [14].

The 10% consists of non-collagenous proteins (Osteopontin OPN, Dentin sialoprotein DSP, DGP, DPP–Bone sialoprotein (BSP), Osteocalcin, Dentin sialoprotein DSP, Dentin matrix protein-1 DMP-1, Collagen type 1, Cbfa1 RUNx2, Bone morphogenetic protein BMP-2, and Transforming growth factor β (TGF-β) 19). Most of the proteins contained in the dentine are also present in bone [15,16,17,18].

One of the earliest studies on the tooth as a biomaterial for grafting is that of Schmidt-Schultz and colleagues, in which using teeth from the late pre-ceramic Neolithic period (approx. 8000 years ago), teeth from the early Middle Ages, and recently extracted teeth, aided in the identification and isolation of growth factors such as Insulin Growth Factor-II (IGF-II), Bone Morphogenetic Protein-2 (BMP-2), and TGF-β [19].

The proteins, quantified and isolated by electrophoresis, were still present undenatured and therefore active even after 8000 years, and the quantity did not differ between the three groups. The results of this study, therefore, reported that the proteins of the extra-cellular matrix of bone and tooth are protected from the aggression of physical and chemical agents even after death by HA, and their osteoinductive capacities can be utilized after resorption of the mineralized part following the regenerative process [20,21].

**Figure 1:**
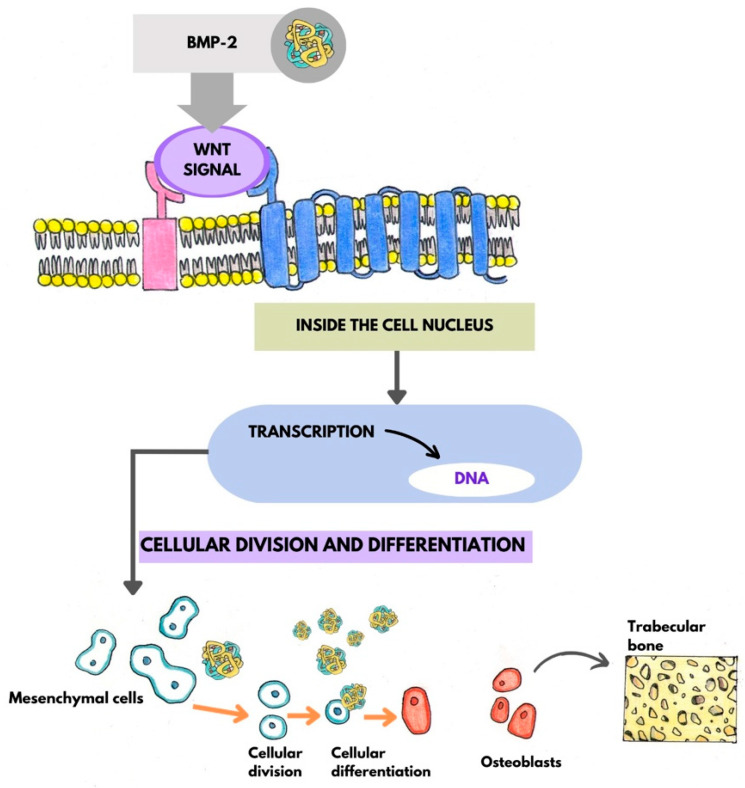
Mechanism of BMP-2, cellular division and differentiation into osteoblasts.

Thirteen types of BMP proteins are recognized. They can activate the process of bone neoformation even in heterotopic locations [22]. Specifically, BMP-2 induces differentiation into the osteoblasts of mesenchymal cells. BMP-3 stimulate bone formation, BMP-7 stimulate differentiation into osteoblasts [23]. BMPs initially activate the replication and migration of mesenchymal cells from surrounding tissues to the regenerated area and then repress the WNT signal that blocks the differentiation of mesenchymal cells [5] (Figure 1).

BMP-2 binds to the cell membrane via the Bone Morphogenetic Protein Receptor Type 1 (BMPR1A), a transmembrane protein of the serine/threonine kinase group, and activates the transformation of mesenchymal cells into osteoblasts that initiate the neo-attachment of bone. BMPs are soluble proteins and require an insoluble carrier, collagen type 1, to activate mesenchymal cells. Individually, the two proteins do not stimulate bone formation [23,24].

IGF-1 and IGF-2 are growth factors activated by Growth Hormone (GH) and are incorporated into the bone matrix. They stimulate cell proliferation and their functions, such as that of collagen type I production. During resorption, they are released, activating osteoblast production and the remodeling of new bone [5,25].

Non-collagenous proteins, which make up 10% of the proteins in dentin, are growth factors that are not specific to the bone but are necessary for its neoformation [26]. OPN controls bone homeostasis, and binding to HA ensures the mineralized base of the bone. BSP stimulates angiogenesis ensuring the neovascularisation essential for the apposition of new bone. Osteocalcin, which indicates bone metabolism, intervenes by activating bone metabolism. A Cbfa Run X2 intervenes in osteoblast differentiation [27,28,29,30].

The mechanisms of stimulation in bone regeneration promoted by demineralized dentin are quite similar to the formation of new bone by autologous bone. After demineralization, both demineralized bone matrix (DBM) and demineralized dentin matrix (DDM) contain type I collagen, growth factors, and bone morphogenetic proteins (BMP-2) [31].

Different techniques for demineralizing teeth were analyzed with equally different results in the production of bone tissue [32]. The degree of sterilization, the repeatability of the system, the liquids and their concentration, the degree of demineralization, the size of the granules, the amount of residual protein after treatment, the wettability and plasticity of the granules, and the ergonomics of the system must be considered when evaluating the various tooth crushing systems [4].

The Tooth Transformer (TT^®^) device proved to be the best-performing system. The need to first prepare the tooth by hand (cleaning and reduction into fragments by milling) and then place it in the grinding container remains a drawback [4]. Intact teeth, devitalized teeth, and even deciduous elements were considered.

Data reported that osteoblasts only adhered to the demineralized surface. Different levels of demineralization are expressed in the Ca/P ratio. The ideal Ca/P ratio in human bone is 1.67 [33,34,35]. A granule size between 400 and 800 µm (constant size in TT^®^) would be the most efficient for the quality and speed of the bone produced (4–6 months) [35,36]. Partially demineralized dentin serves both as a supporting filler and maintains the osteogenic potential of proteins, BMPs, and collagen [37,38,39].

The sterilization of the product and the maneuverability of the six solutions used automatically in sequence by the TT^®^ elevate its quality. The size of the bone defect and the small amount of autologous dental graft available could be a limitation to the use of the system. In this case, either autologous grafts (platelet derivatives together with autologous CGF membranes) and/or xenografts (bioss) or alloplastic grafts (HA or TCP) have been used, exploiting both osteoinductive properties (platelet derivatives and GF) and the scaffold function of the others [39,40,41,42].

The presence of dysmetabolic diseases, such as diabetes, and the increasing age of patients, who often have comorbidities and undergo implant-prosthetic therapies, could affect the bone regenerative response [43]. Recent studies associated the human microbiota with the proper functioning of the entire metabolism and thus a state of well-being. The microbiota refers to several microorganisms larger than the number of cells in the human organism living in symbiosis. They are largely present in the gut (95%), in other parts of the digestive system, in the genital system, and in the eyes and ears, skin, etc. [43,44,45,46].

The genetic composition of the microbiota is called the microbiome [47]. The intestinal microbiota, with its metabolites (short-chain fatty acids SCFA and intestinal wall lipids) interacts with resveratrol (RSV), a naturally-occurring polyphenol, whose anti-inflammatory, anti-cancer, antioxidant and immunostimulant properties improve the clinical consequences of metabolic syndrome, which is frequent in industrialized and developed populations, characterized by obesity, hypertension, hyperglycemia, and cardiovascular disorders [48,49,50,51,52].

The microbiota, with its barrier effect on the intestinal epithelium, reduces the production of inflammatory cytokines and influences the immune system by reducing osteoclastic activity [53,54]. The gut microbiota is also attributed to the ability to produce the hormone IGF-1, which regulates osteoblast differentiation enhanced by the intake of pre- and probiotics [55,56,57]. The microbiota modulates the absorption of vitamins such as folic acid and the vitamin B2–B12 complex. It synthesizes vitamin K, a fundamental carrier of vitamin D [58,59,60]. Vitamin D3 activates TGFβ-1 and TGFβ-2, which are contained in the bone matrix, and which in turn enhance the activation of BMP-2 with an osteoinductive effect [22].

Primary osteoporosis, a major bone disease, appears to be related to the gut microbiota via the gut–brain axis, with mechanisms that are still unclear [61]. In particular, there is an increase in the Dialister and Faecalibacterium genera in the gut microbiota of patients with primary osteoporosis [62]. A valid diagnostic and/or therapeutic guideline in clinical practice could be necessary to monitor the composition of the gut microbiota [63].

The purpose of this study is to evaluate the efficacy of autologous tooth as a grafting material and to investigate its properties and interactions with bone metabolism.

## 2. Materials and Methods

### 2.1. Search Processing

This systematic review has been performed in accordance with the principles of the PRISMA and International Prospective Register of Systematic Review Registry guidelines (ID 390491) [64]. The literature search was performed on PubMed, Cochrane Library, Scopus and Web of Science databases from 1 January 2012 up to 22 November 2022, with English-language restriction. A combination of words that matched the purpose of our investigation, whose primary focus is the use of grafts with autologous materials of dental origin was used; hence, the following Boolean keywords were chosen: (“demineralized dentin matrix” OR “tooth graft”) AND “biomaterial”.

### 2.2. Inclusion Criteria

Reviewers worked in duplicate and analyzed all suitable trials, considering the following inclusion criteria: (1) studies only on humans; (2) open-access studies; and (3) studies that analyzed the use of grafts with autologous materials of dental origin, including articles in which this is associated with other materials and possible associations with systemic diseases, microbiota or intake of RSV, curcumin and quercetin. Articles dealing with dental grafts using non-autologous materials were excluded, as were articles in non-English languages.

## 3. Results

**Figure 2:**
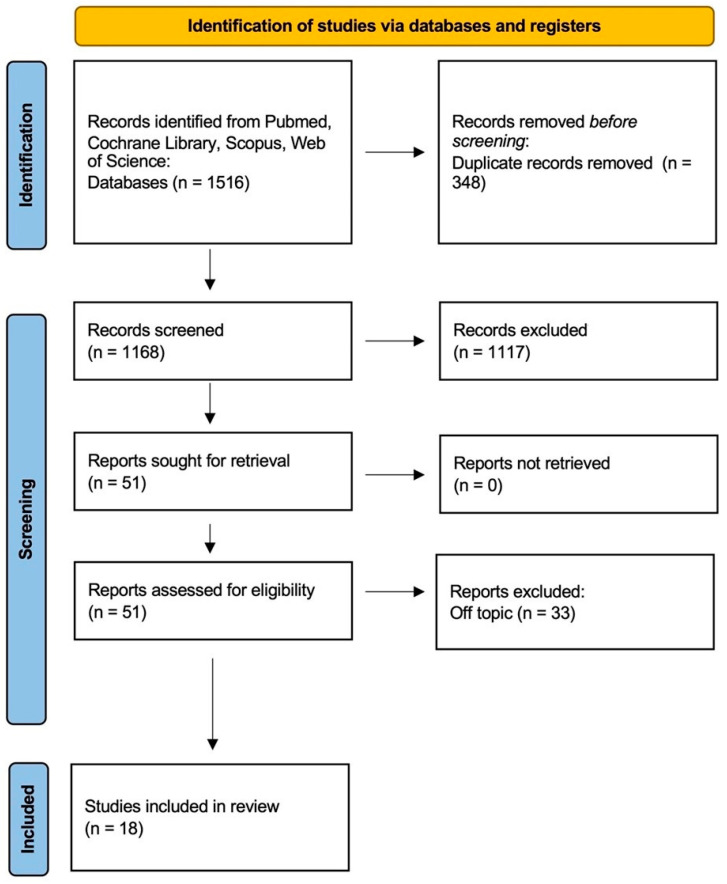
PRISMA flowchart diagram of the inclusion process.

A total of 1516 articles were identified from Pubmed (755), Scopus (537), Cochrane Library (85), and Web of Science (139) databases, which led to 1168 works after removing duplicates (348). Nineteen relevant publications were added by searching the reference list of eligible papers. A total of 1117 articles were excluded by analysis of the title and abstract. The remaining 51 articles were added to the 19 papers found by reference list, leading to 70 publications that were assessed for eligibility by the authors. A total of 33 publications were excluded because they were off-topic. A final 18 studies were included for qualitative analysis of the review (Figure 2) (Table 1).

## 4. The Autologous Tooth Graft

Autologous bone grafts have been the gold standard for bone defect regeneration for more than a century, having an important impact on regenerative medicine. Despite the diversity of structure between dentin and cortical bone, the biochemical composition presents similarities. The majority of dentin is composed of proteins common to both dentine and bone. Collagen guarantees resilience and elasticity, making them resistant to fractures. The 10% is made in Non-Collagenous Protein [15,16,80].

Bone induction is a biological process based on the recruitment of undifferentiated and pluripotent native cells which develop into the bone-forming cell pathway. The stimulation of new bone formation by bone autograft is quite similar to the bone regeneration favored by demineralized dentine [5]. These dynamics consist of a complex mechanism of cellular differentiation induced by the interaction of inducing cells and responding cells that stimulate new bone formation. Microscopically, dentin is reabsorbed thanks to the increase in the number of cell mesenchymal, macrophages, and osteoclasts carrying collagenolytic enzymes [5].

It has been demonstrated that the use of demineralized dentin induces invasion of the dentinal tubules by the processes of osteocytes and their cytoplasm [81]. The demineralization process of dentin increases BMP-2 bioavailability, leading to a new osteoinductive bone. In addition, the demineralization process in enamel, which is even less effective than in dentin, gives an increase in BMP-2 bioavailability and improved performance in vitro compared with native enamel. In this study, human dentin and enamel were reduced in demineralized and sterilized particles with a diameter of 1000 µm, [81]. Bono et al. demonstrated that sterilization is a fundamental process for grafts, even more so than the other chemical and physical treatments, and demineralization improves the osteoinductive properties of dentin [68,82].

A radiographic one-year follow-up evaluation of two case reports showed an increase in radiopacity and density on the site where a demineralized dentin graft had been used, resulting in complete restoration and healing via new bone formation without any clinical complication for the patient [31]. In another case report, a 1-year intraoral radiological evaluation enabled to appreciate stability of marginal bone levels where a conical-shaped implant has been positioned 24 weeks after tooth extraction filling the socket with dentin graft. CBCT showed that a new mineralized bone formed. The histological evaluation of the same case showed that spaces between root fragments of the tooth and bone are filled with connective tissue and the new bone fits tightly with both cementum and dentin [70]. Autogenous teeth grafted as particles were gradually reabsorbed and replaced by new vital bone thanks to osteoinduction and osteoconduction, leading to good implant primary stability. Histological analysis demonstrated that more than 64% of the surface of the implant is in direct contact with bone [78]. There was no difficulty with implant osseointegration thanks to the lack of residual dentin particles that were never in contact with the implant surface. The healing process of the autogenous tooth-derived graft was well harmonized with implant osseointegration demonstrated by a continuous dense mineralized zone of two to five cell layers with osteoblasts that surround the implant surface [78].

Minetti et al. evaluated the histo-morphometric results (new bone in upper jaw sites 37.9 ± 21.9% and new bone in lower jaw sites 38.0 ± 22.0%) of the application of autogenous tooth-derived bone substitute material in patients treated with alveolus preservation. A total of 101 histological specimens were analyzed by evaluating the total amount of bone, residual dental graft material, and viable bone. The authors stated that alveolar socket preservation employing demineralized autologous tooth-derived biomaterial was a reliable method to obtain fresh vital bone to sustain dental implant rehabilitation [76].

In the study of Sang-Ho Jun et al., tooth bone graft (size 500–1000 µm) and inorganic bovine bone (Bio-Oss) were used as sinus bone grafts in patients with residual bone height less than 500 µm in the maxillary posterior area. After four months the healing state of bone graft sites was evaluated. In a microcomputed tomography analysis, there was no difference in bone density and height between the two groups, instead, in the histo-morphometric analysis, there was a significant difference in the osteoid thickness of the two groups [66].

DDM (size 500–1000 µm) was compared with Bio-Oss as a bone substitute in Guided Bone Regeneration (GBR) in two groups of patients with periodontal teeth and need of implant rehabilitation. GBR was performed with the use of collagen membrane and contextual implant placement. The implant stability, evaluated by Osstell Mentor, and marginal bone resorption, evaluated with x-ray examinations, were measured at T0, at 6 and 18 months after surgery. The values of these parameters were superimposable in the two groups [69].

Minetti et al. studied the use of DDM (406 µm and 815 µm with peaks up to 1110 µm) as a bone substitute for alveolar ridge augmentation with and without collagen membrane. At four months, a bone tissue biopsy to perform and histological analysis showed more bone volume and vital bone in the sites where membrane had been used in association with DDM [79].

DDM can also be used in association with a collagen membrane in post-extraction site preservation and it can be produced from vital or endodontically treated teeth. All filling materials (gutta-percha, cement, etc.) were removed from the teeth. After four months, bone biopsies were performed. The histological and histo-morphometric analyses showed that there was no significant difference in the bone regeneration of sites where DDM was obtained from vital or endodontically treated teeth [37].

Preservation of the post-extraction site of the lower third molar, with tooth bone graft (300–1200 μm) included, ensured bone regeneration of the site and reduced the periodontal defect on the distal side of the lower second molar, which can be created at the site that is left to heal spontaneously [73].

A retrospective study conducted by Korsch and Peichl analyzed the use of autologous dentin for lateral ridge defect reconstruction (tooth-shell technique). This procedure was compared to the bone-shell technique on autogenous bone, according to Khoury. In both groups, implants were placed simultaneously, follow-up was performed at three-month intervals and prosthetic restoration after five months [74]. No significant differences were found between the two groups in terms of complications and outcomes. The integrity of the buccal lamella was preserved in all implants, and all implants had fully osseointegrated, suggesting the use of autologous dentin as a valid and less invasive alternative to autologous bone [74].

In another study, demineralized human dentin and implants were simultaneously implanted in the region of bone defects. No intraoperative or postoperative complications occurred, and no bone resorption was observed. There were no implant or abutment failures in the subsequent 35 months [65].

Kim et al., in a case series, suggested the use of an autogenous fresh demineralized tooth prepared at chairside immediately after extraction for socket preservation. They found that socket preservation using powder, chip, or block type Auto-FDT with a stress-shielding barrier membrane was successful to retain the ridge heights and widths needed for implants [67].

From the data emerging from the histological examinations of Minetti et al., the average vital bone newly formed following the regeneration process using tooth-derived grafts was approximately 27%. This value was significant. A very high bone volume can mean bone containing only graft material that cannot guarantee osteointegration of implants. On the other hand, a high percentage of vital bone indicated a large rate of truly newly formed regenerated vital bone that will participate in the metabolism dynamics and therefore will allow stability and integration [5].

Okubo et al. presented a case of alveolar bone preservation using autogenous primary dental material, with clinical and histological results. The main advantages of the presented technique were great availability in terms of volume, the use of fully autogenous material to reduce the possibility of adverse immune reactions in case of refusal of the patient to receive biomaterials of animal origin, the use of deciduous tooth is completely free of any biological or economic cost, and deciduous teeth, having less enamel than permanent teeth, have greater osteoinduction [77].

In 2015 Park et al. conducted an in vivo study on the use of deciduous teeth reduced to powder and used as filler material after appropriate demineralization, concluding that deciduous teeth had adequate structural and physicochemical characteristics suitable to be used as grafting materials [83].

In 2018 Bono et al. described that deciduous teeth could be used as grafting materials in bone augmentation treatments. They also shed light that collagen and BMP-2 protein contents in demineralized tooth material were conserved after chemical treatment. Furthermore, they assessed in vitro the response of osteoblastic cells to exogenous stimulation of BMP-2 (at different protein concentrations) to detect the minimum concentration of BMP-2 able to induce the expression of alkaline phosphatase, the marker of early osteoblastic phenotype [68].

A case report, with two-year follow-up, exposed that socket preservation was possible using dental grafting material, and the same site was subsequently treated with implant-supported rehabilitation [72].

Bone remodeling is a constant and complex process for bone tissues in the body and could cause resorption of alveolar bone when a tooth is no longer in place. To compensate for insufficient available bone volume, a number of surgical procedures designed to augment bone volume are described and validated in the scientific literature [72]. As we know from the literature, after tooth extraction, healing of the socket happens through the sequence of many steps, beginning with the stabilization of the clot, the formation of fibrin, and, finally, the recruitment of osteoblasts that will be in charge of the formation of new bone. Several biomaterials and techniques for alveolar volume preservation have been reported, with many functionalities (osteoconduction, osteoinduction, or even stimulation) according to the features of each material [72].

In several clinical situations, autogenous bone grafting has been recognized as the most successful biomaterial. Human dentin and bone exhibit many similarities in regard to mineralization. In fact, after demineralization, both DDM and DBM are made up predominantly of type I collagen (90%) and NCP and, among them, growth factors; therefore, human DDM is a bio-collagen scaffold containing osteoinductive growth factors that supply an appropriate setting to promote new bone formation [72].

According to the study of Cervera-Maillo et al., on 10 partially edentulous patients, a particle-sized dentin graft was performed as an alternative material for socket preservation, split technique, and maxillary sinus elevation. After 24 months of evaluation there was a high bone resorption rate and bone replacement without inflammation, suggesting this as an acceptable biomaterial for several bone defects due to its osteoinductive and osteoconductive properties [75].

Open tubes of dentine particles allowed capillaries to enter, favouring rapid resorption. Clinically and histologically, dentin graft performance is at least comparable to xenogeneic or allogeneic biomaterials [75].

The study of Minetti et al. evaluated the use of the extracted tooth as an autologous graft for socket preservation on patients with post-extraction bone defects. For this purpose, The TT^®^ shredding and decontamination machine was used. The graft thus obtained was subsequently inserted at the time of extraction [38].

An innovative preparation method was used, using the dedicated automated TT^®^ device, capable of transforming autologous teeth into suitable grafting material. The extracted tooth was cleaned and treated with a dental transformer and socket preservation was performed. Thirteen biopsies were performed to analyze the histological findings over an average time of four months to evaluate if the autologous tooth graft obtained from the root after endodontic therapy should be used in human bone regeneration as a graft for the placement of dental implants [38].

The study of Kadkhodazadeh et al. aimed to evaluate the osteopromoting ability of human tooth powder and compare it with a bovine xenograft, a synthetic material, and a demineralized freeze-dried bone allograft (DFDBA). Tooth powder was able to increase cell proliferation compared with bovine xenograft, synthetic graft, and DFDBA. However, its osteopromotional capacity was inferior to that of osteogenic materials [71].

The quantity of grafting needed in cases of severe bone deficiencies is a source of discussion. Depending on the tooth, the mean weight and volume obtained by grinding the tooth with TT^®^ varied between 0.68 g and 1.88 g and 0.38 cc and 0.96 cc, respectively [5].

In a critical size defect in rats, dual administration of the angiogenic growth factors VEGF and osteogenic BMP-2 demonstrated a remarkable capacity for almost full regeneration [84]. Different studies that evaluated the socket preservation technique with dentin particulate autograft and platelet-rich fibrin concluded that this technique and biomaterial combination had produced well-maintained vertical socket dimensions and minimal horizontal ridge reduction [85,86]. Platelet-rich fibrin is bioactive molecules that can increase cell proliferation and bone remodelling [87,88]. The fibrin matrix’s architecture affects the trapping/release of *GF* [89] Within 7–14 days, platelets and macrophages release a variety of growth factors, such as vascular endothelial growth factor (VEGF), platelet-derived growth factor (PDGF), transforming growth factor-β1 (TGF-β1), and insulin-like growth factor (IGF) [89,90,91,92,93].

When there are not enough teeth, Umebayashi et al. recommend using autologous bone or the combination of autologous tooth and other biomaterials [39]. In their study, the autologous tooth matrix was partially demineralised and combined with cancellous and medullary bone particles for bilateral sinus lift and anterior maxillary reconstruction. This combination showed osteoinductive and osteoconductive capabilities in bone regeneration [39].

In another study, partially demineralised dental matrix was combined with xenograft material (Bio-oss) obtaining good results in bone regeneration, even if bovine bone resorpition was slower than with the dental matrix [40]. In extensive bone deficiencies, the dental matrix can be coupled with other biomaterials to produce excellent results. [39,40,41,42].

## 5. Devices for Tooth Processing

The material of the tooth can be considered an ideal scaffold due to its osteoconductive properties, which come from its construction with HA and collagen types 1 and 3, and its natural protein content also makes it a material with osteoinductive properties [4,15]. However, this property depends on the amount of proteins that will be found in the material after treatment; therefore, the way of processing this material is very important and still under discussion. Finding a quick, easy procedure that can demineralize, cleanse, and crush teeth while preserving all of their natural properties is the real challenge [5].

**Figure 3:**
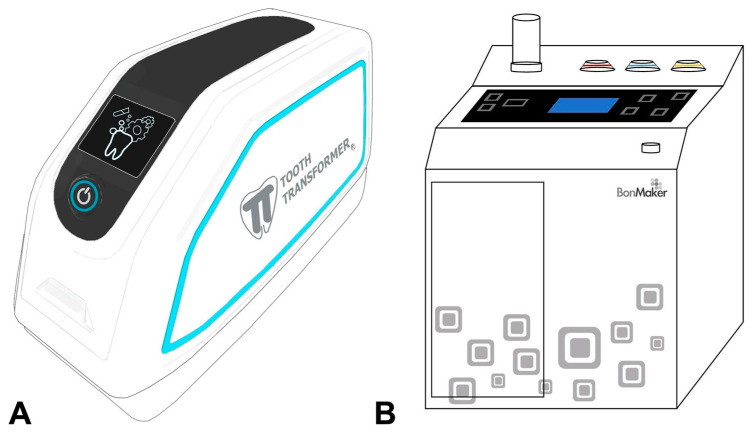
(**A**) The Tooth Transformer^®^ (CE); (**B**) BonMaker^®^ (CE).

In the market to date, there are two devices suitable for using the tooth as a grafting material that have medical CE markings: TT^®^ and Bon Maker^®^ (Figure 3) [68,94]. Two other devices, Kometabio and Vacuasonic, do not have the medical CE Marking and therefore have not been considered.

### 5.1. Step 1: Tooth Cleaning

**Figure 4:**
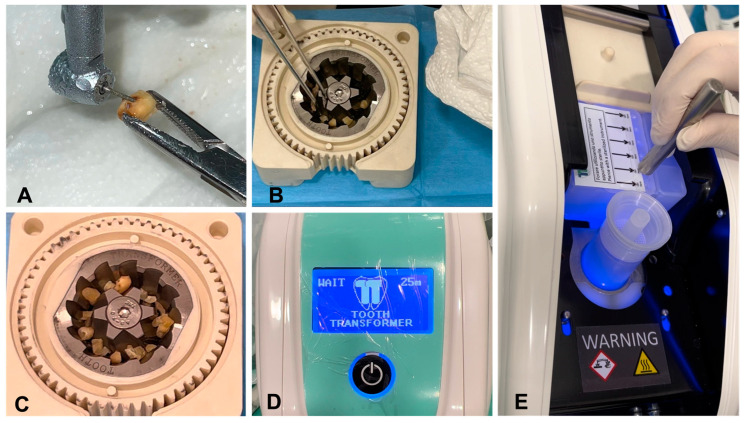
(**A**) Extracted teeth are cleaned with the use of a diamond bur mounted on a turbine to remove any carious process residue from the tooth surface; (**B**) Dental elements prepared for grinding; (**C**) Grinding with tooth fragments positioned in Tooth Grinder^®^ ready for further shredding; (**D**) Inserted the liquids and pierced the cartridge before the beginning of the transformation cycle; (**E**) Starting the shredding process with tooth grinding. The transformation cycle lasts only 25 min.

After extraction, caries, tartar, soft tissue debris, fillings, cements, and prosthetic parts must be removed from the tooth (Figure 4A). Teeth with root canal therapies can also be used. For the Bon Maker^®^ device, which uses a sieve to separate granules according to size, it is advisable to remove with tweezers the parts that are not consistent with the tooth tissue after grinding. For the TT^®^, which requires sectioning of the tooth for the trituration step (Figure 4B), cleaning from residual root canal care can be performed during the sectioning stage, simplifying the cleaning procedure for small sections that are more easily visible using magnifiers [5].

### 5.2. Step 2: Tooth Grinding

The Bon Maker^®^ device uses a hammer and pestle to crush the tooth, a risky procedure for the operator. Having performed the crushing, the tooth is placed in a non-sterilizable high-speed mill and the granules are separated by a manual sieve, which separates fragments of different sizes by making use of two nets of different filtrations: the larger granules, of 850 μm, are blocked by the first filter, while the finer granules, of 450 μm pass, through the second filter, into the lower plate [5,94]. The TT^®^, although it has the disadvantage of not being able to insert a whole tooth into the shredder, is equipped with a multi-purpose sterilizable system that works at low speed, which makes it possible to avoid the loss of tooth substance in pulverization [5] (Figure 4C).

### 5.3. Step 3: Treatment by Device

#### 5.3.1. Bon Maker^®^

The granules should be manually placed in a sterilizable plastic cylindrical container (Bonbin), which should be inserted into a slot in the machine’s upper front. The liquids, which are contained in disposable flasks, must be manually emptied into their respective cavities according to a color code. A bottle to be filled with saline solution and screwed onto the device’s top is also required. The material is extracted from the Bonbin at the end of the treatment, which takes about 26 min. The spent and contaminated liquids are collected in a flask glass that is placed behind a door at the front of the device and must be emptied after a few uses. The liquids’ composition was analyzed at the Politecnico di Milano, with the following results: HCl 0.45 M-H2O2 130 volumes-ethanol 62.6% chloroform 31.3% water 6.1% + saline wash solution; HCl 0.56 M-H2O2 120 volumes-ethanol 47.2% chloroform 47.2% water 5.6% + saline wash solution [5,95].

#### 5.3.2. Tooth Transformer^®^

After inserting the tooth into the shredder, the device is closed and inserted. A liquid cartridge and a cylinder with a granulate collection cup (maker) are inserted into the device in their respective housings, the cartridge is activated by punching, and the process begins when the door is closed and the button is pressed (Figure 4D). The liquids are three distinct solutions contained in six distinct compartments of the same single-use cartridge. Two of them are active liquids made up of 0.1 m hydrochloric acid and 10% hydrogen peroxide, while the other is demineralized water. The four compartments filled with mineralized water are employed to eliminate the acid residues in four separate method steps. Following the automatic perforation of the lower cartridge membrane, the six liquids in the cartridge fall by gravity and initiate the process. The procedure is completely automated and repeats the same steps every time. The transformation cycle lasts only 25 min (Figure 4E). The granules are dropped into the collection basket during the first phase of low-speed shredding. To avoid protein damage, the granules are subjected to UVA rays and ultrasonic vibrations with temperature changes, always below 45 °C. At the end of the process, the used and contaminated liquids remain inside their container and can be removed, as they are disposable. [68,96,97,98].

## 6. Tooth Transformer^®^ Technique

TT^®^ technology allows for obtaining a demineralized and disinfected tooth material with a pasty consistency [5,94].

**Figure 5:**
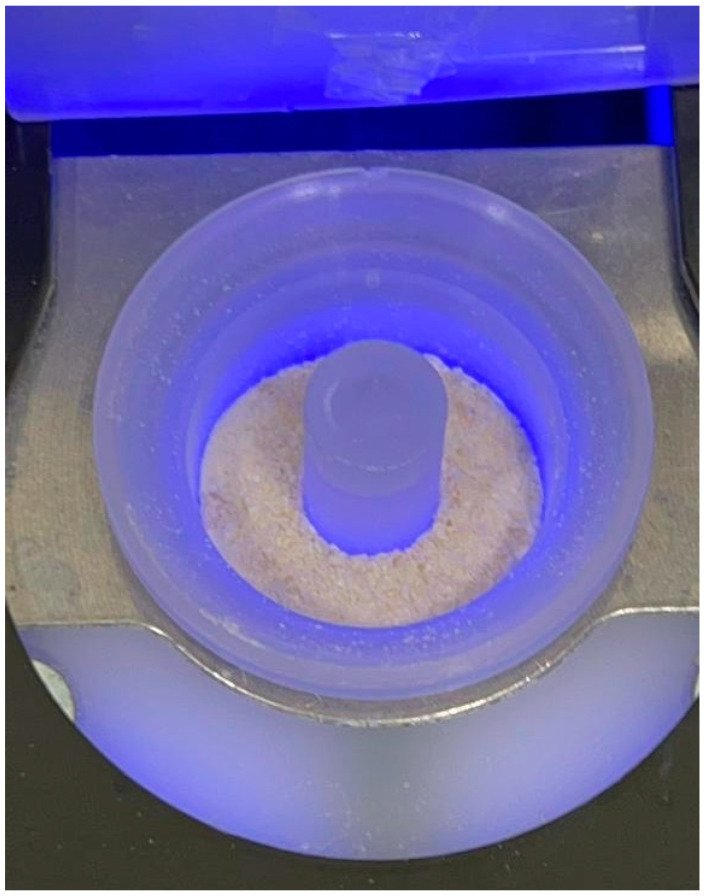
Term processing of the teeth that are shredded between 400 and 1000 µm. Ready for grafting in the oral cavity.

The processing of the tooth material with TT^®^ makes it possible to obtain a 400–800 µm graft at the end of the procedure [5,99] (Figure 5). Different studies have been conducted on the size of the graft particles and the impact they have on bone formation. It is said that the graft with larger particle size favors the growth of blood vessels as it leaves more space [100]. Dozza et al., in his study with three different sizes of DBM particles, suggested using an average size of 0.5 to 1 mm for a better result [34]. Koga et al. performed a comparative study between DDM, partial DDM, and mineralized dentin matrix using three different sizes of graft particles in each group [35]. According to this study, partial demineralization of the dental matrix with particles between 500 and 1000 µm has a larger potential for regeneration than total demineralization since it retains more growth factors that could influence osteogenesis [34,35]. The demineralized dental matrix resorbs faster than mineralized dentin since the biodegradation of big particles with a high crystalline content is quite impossible. The best osteoconduction results come from small-sized HA crystals; however, very small parts could lead to rapid resorption of the graft and failure to preserve the volume [5,36,37,101] TT^®^ has two different speed grinders, the low speed and the high speed. this grinding procedure offers a particle size of the graft between 400 and 800 µm [5]. Scanning Electron Microscope (SEM) analysis revealed that the autologous dental graft’s density, roughness, and homogeneity are roughly equivalent to those of autogenous cortical bone, with a surface that contains both mineralized material and organic parts. [36]. The tooth matrix does not lose bone volume over time like other autologous grafts do [101].

The porosity has an impact on the graft’s early fibro-vascularization and new bone replacement. Additionally, the scaffold’s pore size has the power to influence how osteogenesis develops. Bigger scaffold pores enable vascularization, which directly signals the start of osteogenesis [102,103]. Dentin is formed by microtubules that are created as a result of the passage of nerve extensions. These tubes have a diameter that varies from 2.5 µm to 0.9 µm with an average of 1.2 µm [104]. The macrophages, mesenchymal cells, and osteoclasts must increase to determine collagenolytic enzyme production so that the resorption process induced by the biomaterial graft begins [105]. Mesenchymal cells, osteoblasts, and other bone cells range in size from 10 to 20 micrometers, while osteoclasts range in size from 20 to 100 µm or more [106,107]. Cell invasion into the dentinal tubules is not conceivable and the demineralization, by increasing the size of the dentinal tubules, favors the adhesion and the activity of the osteoblasts and their number. Resorption of undecalcified dentin is incomplete or delayed [108]. Minerals seem to make the matrix inaccessible to the action of collagenolytic enzymes. Under the microscope, non-demineralized dentin appears with tubules blocked by mineralized tissue material [109]. Bertassoni et al.’s findings demonstrated that the dentinal peritubular is made up of a collagen-free organic network that is embedded with HA. After acid treatment, the mineral was primarily dissolved by demineralization in peritubular dentin, which had expanded the tubules as well as a meshwork that protruded toward the lumen of the tubules was seen. After demineralization, a dense network of collagen fibrils with amorphous molecules located in it was distinguished [110]. For this reason, undecalcified dentin was not resorbed until 8–12 weeks, much later than decalcified dentin. A notable element is that after one, two, and three weeks of demineralization, the tubule widths in the wet state—which were 1.3 ± 0.2 μm in sound dentine—are 2.5 ± 0.3 μm, 2.2 ± 0.3 μm, and 1.7 ± 0.2 μm, respectively [111]. However, a varied image of the surface with a higher or lesser openness of the tubules is obtained depending on the liquid or combination of liquids utilized. It is believed that a biomaterial’s submicronic surface structure encourages osteogenesis by controlling the Transforming Growth Factor (TGF) pathway and causing Mesenchymal Stem Cell (MSC) differentiation [112]. Tanoue et al.’s study showed that osteocyte processes and cytoplasm invaded the demineralized dentinal tubules. The degree of invasion was 5 μm away from the new DDM bone contact and osteocyte cellular processes penetrated the dentinal tubules, forming bone tissue that filled the DDM surface [81]. Koga et al. cultivated osteoblasts on the surfaces of mineralized dentin and demineralized dentine matrixes, and only the demineralized dentine matrix surface showed evidence of osteoblast adhesion, this was not observed in mineralized dentin [35]. Therefore, mineral traces on the surface, which correspond to high Ca and P values may limit the ability of the cells to adhere. Different levels of demineralization lead to different concentrations of the Ca/P ratio. The ideal Ca/P ratio in human bones is 1.67 [33]. The TT^®^ technique also protects the proteins in the tooth matrix while providing a Ca/P ratio (1.70) that is closer to the natural ratio observed in bone [5]. In contrast to the non-demineralized matrix, this partial demineralization of the tooth obtained from TT^®^ decreases the concentration of Ca and P while simultaneously increasing the bioavailability of BMP-2 [68,113]. Bono et al. supported the idea of employing demineralized, treated teeth by providing evidence that BMP-2 is more bioavailable after TT^®^ demineralization. Six solutions (liquids) are used in the process to demineralize and disinfect the crushed tooth, giving a pasty consistency [68]. Increased wettability and increased hydrophilicity of the surface are two key features of this surface modification. In comparison to hydrophobic surfaces, hydrophilic surfaces have shown enhanced mineralization, local factor generation, and osteoblast maturation. Microtomography affects osteoblast maturation [114,115]. Some studies claimed that demineralization damages the microstructure of dentin and eliminates the organic material found in it [116,117]. The protocol used by TT^®^ with six liquids does not damage the dentinal structure, but BMP-2 availability in the matrix is improved [5,68].

High demineralization substances result in graft materials with low osteogenic potential since they reduce or eliminate the BMP-2 and other proteins found in teeth [5], Such as those in the small integrin-binding ligand N-linked glycoprotein (SIBLING) family [118]. Natural cell-adhesive and Matrix Metalloproteinases (MMP) binding sites that are crucial for viability, proliferation, and differentiation are retained by the dentin matrix proteins [119]. DMP1, also known as AG1 earlier, was first discovered in teeth but was later discovered in bones, where it is mainly expressed by osteocytes. DMP1 is a multifunctional protein that plays roles in the development of odonto- and osteoblasts, phosphate homeostasis, and the biomineralization of bones and dentin [120,121,122,123,124,125].

The development of mesenchymal cells into osteoblasts is greatly aided by the BMPs, which belong to the TGFbeta superfamily [27]. Recombinant human BMP-2(rhBMP-2) was authorized for use by the Food and Drug Administration (FDA) due to its range of uses and osteogenic potential. BMP-2 binds to type I and type II serine/threonine kinase receptors on target cells. This process activates the Smad (canonical) and non-Smad (non-canonical) signaling pathways, which in turn activate osteogenic genes like Osterix and RUNX2. This process stimulates MSC differentiation into osteoblasts [28]. Li et al. detected an increase in the activity of Alkaline Phosphatase (ALP) which results in an increase in osteodifferentiation activity by the BMP-2 [29]. After undergoing TT^®^ treatment, the concentration of BMP-2 in the dentin matrix is 22 ng/mL, which is higher than the minimum concentration of 12.5 ng/mL required to cause considerable ALP activity, as described by Blum et al. [5,113,126].

The tooth material processed with the TT^®^ technique was applied to fill the alveoli to preserve the bone after tooth extraction (socket preservation technique) [5]. According to the authors, the success rate of the implant following bone regeneration was 99.1%, and it took four months for the bone to repair and be prepared for the implant. BMP-2 and collagen are not removed by the solution’s demineralization and decontamination processes. [37,38]. By serving as a carrier for the BMP-2 present in the dental structure, demineralized dentin combines the characteristics of the scaffold with those of the BMP-2. However, substances that cause high demineralization produce dental-derived graft materials with low osteogenic potential because they reduce or eliminate the proteins present in the tooth, including BMP-2.

## 7. Microbiota and Bone Metabolism

Systemic health is the result of the equilibrium between the various components of the gut. The intestinal environment represents 95% of the microorganisms present in the body. The remainder is present in other environments (for example: mouth, nose, skin, and genitals) [43,44,45].

The main families of bacteria that constitute the gut microbiota are Actinobacteria, Bacteroides, Firmicutes, Proteobacteria, and Verrucomicrobia. The confirmation of the intestine’s neuro–endocrine–immune nature over the past 20 years has been one of science’s most important discoveries, known as the gut–brain axis [46,127,128].

There has been much research and discussion on the significance of the gut microbiota in the bone regeneration process [61,129,130,131,132,133,134,135,136]:

- The intestinal microbiota can regulate bone density because it increases the solubility of inorganic salts (calcium, phosphate, and magnesium) and the absorption through the intestinal wall [137,138];
- Gut microbiota homeostasis increases calbindin-D9k expression resulting in increased calcium reabsorption [139];
- The intestinal microbiota regulates the synthesis of serotonin and therefore bone metabolism [140];
- The homeostasis of the intestinal microbiota promotes the proliferation of enterocytes, strengthening the absorption of minerals [141];
- The microbiota ensures the barrier effect of the intestinal epithelium, which prevents “leaky gut” by avoiding the increase in inflammatory cytokines which induce activation of osteoclasts and bone destruction [142,143];
- The intestinal microbiota produces SCFA: SCFAs inhibit nuclear factor kappa B (NF-kB) and therefore inflammation. Furthermore, it reduces the expression of TNF Receptor Associated Factor 6 (TRAF6) and Nuclear Factor Of Activated T Cells 1 (NFATc1) by inhibiting the action of osteoclasts [144,145];
- The intestinal microbiota promotes the production of the hormone IGF-1, which induces the differentiation of osteoblasts [146];
- The intestinal microbiota interacts with the immune system by promoting the production of proinflammatory cytokines with an osteoclastogenic action, determining bone resorption [53,54].

The comprehension of the relationship between gut microbiota and bone mass disorders will be made easier with a greater knowledge of how microorganisms’ shape and function change [147,148]. It is still unknown how the gut flora alters in people with osteoporosis. Most likely, the immune–inflammatory axis may serve as a crucial link connecting bone metabolism to the gut flora. According to studies, mice grown germ-free (GF) have more bone mass. In the bone and bone marrow of GF animals, the scientists found fewer osteoclasts, osteoclast precursor cells, CD4 (+) cells, and inflammatory cytokines [149]. Additionally, they claimed that once GF mice had their gut flora transplanted, their bone mass returned to normal [133].

Moreover, studies by Bindels et al. (2015) [150], Maekawa and Hajishengallis (2014) [151], and Scholz-Ahrens et al. (2007) [55] have demonstrated that taking specific pre- and probiotics might enhance bone mass. According to research, gut microbiota and some probiotics may control IGF-1, TNF-, and IL-1, altering bone development and formation [56,152].

Additionally, the intestinal system allows vitamins like folic acid and the vitamin B2-12 complex absorption, permits the synthesis of vitamin K, which is crucial for the vitamin D absorption mechanism, and plays a crucial role in the homeostasis of the skeletal system and in the development of osteocytes, osteoblasts, and osteoclasts [58,59,60]. SCFAs like acetic acid, which are the sources of energy for both the complex cellular structure that makes up the intestinal epithelium and for the ongoing maintenance of the skeletal system, are produced by the intestine microbiome [57,153].

The link that exists between the bone system and dysbiosis is yet to be investigated. It seems that in a state of dysbiosis, bacterial lipopolysaccharide (LPS) endotoxin increases, promoting endotoxemia and osteoclastic activity [61,129]. The osteoblast and other cells express the androgen receptor (AR) and the estrogen receptor (ER). The effect of estrogens and androgens on bone mass is influenced by osteoclast progenitors [154]. Estrogens may have a protective effect on the maintenance of cortical bone mass as a result of ER signaling that is not nuclear-started. The ER in mesenchymal cells may promote bone apposition from the periosteum [155]. Recent studies demonstrated that the activation of the RANK Ligand/System RANK/Osteoprotegerin (OPG) is partially responsible for the adverse effects of estrogens and their deficit in bone remodeling [43]. Mature osteoclasts convert mononuclear precursors depending on how the RANKL and RANK interact. Because estrogen levels are lower in postmenopausal women, osteoblasts express more RANKL at higher rates, which encourages bone resorption [156,157].

The few results present in the literature showed a distinct kind of bacterium: patients with osteopenia showed an increase in the Firmicutes phyla and a decrease in the Bacteroidetes phyla compared to the control group [152]. Furthermore, subjects with a chronic inflammation (subjects affected by Crohn’s disease and ulcerative colitis) present a picture of osteoporosis [158]. Hormones responsible for the production and stabilization of bone tissue are inhibited by the chronic overexpression of pro-inflammatory interleukins, causing the development of osteoporosis [152,159]. It is now known that the microbiota plays an important role in many systemic conditions; therefore, intestinal and oral dysbiosis influence osteoporosis and bone loss [160,161]. Probiotics interacting with intestinal and oral microbiota give good results in various conditions and pathologies [47,162,163,164]. With the use of probiotics for oral administration, the control of intestinal microbiota is a crucial component in the immune system’s response [165]

Nowadays literature confirms the correlation between general health and gut microbiome which focuses on T- and Th17-immune cells. Therefore, dysbiosis can affect the immune system necessary for bone balance. Thus, the therapy of excessive bone resorption and bone healing disorders may benefit from gut microbiota homeostasis [53].

## 8. Conclusions

In many respects, autologous bone grafts for bone regeneration continue to be the gold standard of bone grafting materials. Demineralized dentin can be used as a grafting material, demonstrating high cell compatibility and rapid bone regeneration due to an optimal balance between resorption and production of newly formed bone. In the alveolus preservation technique, the use of demineralized dentin and platelet-rich fibrin, in combination with biomaterials, allows good vertical preservation of the alveolus with minimal horizontal reduction. Platelet-Rich Growth Factor (PRFG), Platet-Derived Growth Factor (PDGF), and Endothelial Growth Factor (VEGF) promote neoangiogenesis and morphogenesis when mixed with bone graft substitutes. The proper process of dentin demineralization allows increased bioavailability of BMP-2 with osteoinductive as well as osteoconductive capabilities. The TT^®^ is a device with system repeatability capabilities: sterilization, automatic distribution of fluids and their concentration, degree of demineralization, constant granule size (400–800 µm), wettability and plasticity of granules, and preservation of BMP-2. The intestinal microbiota interacts with RVS due to its anti-inflammatory and antimicrobial properties by modulating the absorption of vitamins (folic acid and vitamins B2–B12). It synthesizes vitamin K, a key carrier of vitamin D. This study highlights some correlation between bone and gut microbiota, although the link between bone metabolism and dysbiosis needs further study. Advances in tissue engineering and future research should produce more meaningful scientific evidence to understand the efficacy of these treatments and spread into daily clinical use and not be limited to scientific research alone.

## Figures and Tables

**Table 1:** Descriptive summary of item selection.

Authors (Year)	Type of the Study	Aim of the Study	Materials	Results
Tazaki et al. (2012) [65]	Clinical report	The use of autologous dentin grafts for the treatment of bone defects.	The extracted tooth was crushed by a newly developed automatic mill. The crushed granules were demineralised in 2% HNO3. The granules were washed in distilled cold water and freeze-dried (size: 0.5–2.0 mm).	The human dentin can be used as an autogenous biomaterial for local bone engineering.
Kabir et al. (2014) [31]	Clinical report	These studies imply that dentin may actually replace bone as a viable biomaterial.	Case 1: a 29-year-old male patient. A #38 was used to create tooth-derived granules, which were then demineralized in 2% HNO3 for 30 min and thoroughly cleaned. DDM was transplanted into the bone gap. Case 2: A 20-year-old woman who had an impacted third molar (#48). The DDM autograft and extraction of the affected tooth were completed concurrently.	90%–95% of patient-own recycled dentin matrix had remodeled into bone, resulting in excellent bone defect repair.
Jun et al. (2014) [66]	A prospective clinical study	Evaluation of DDM and Bio-Oss as sinus bone grafts in patients with residual bone height less than 5.0 mm in the maxillary posterior area.	43 patients, 21 in the control (Bio-Oss) and 22 in the test group (DDM 0.5–1.0 mm). After four months the sites were analyzed with microcomputed tomography analysis and histomorphometric analysis.	In the two groups, there was no difference in bone density and height, instead, there was a significant difference in osteoid thickness.
Kim et al. (2015) [67]	Case reports	To evaluate the clinical use of the chairside-prepared demineralised tooth immediately after extraction for alveolus preservation.	The use of the extracted tooth as graft material radiographic and histological evaluation of the graft site.	The use of the dental block is effective in maintaining height and thickness of the bone in the preservation of the alveolus.
Bono et al. (2017) [68]	In vitro study	To examinate the effects of demineralization on the physical–chemical and biological behavior of D and E.	Human dentin and enamel were minced into particles (Ø < 1 mm), demineralized, and sterilized. Thorough physical–chemical and biochemical characterizations of native and demineralized materials were performed by SEM and EDS analysis and ELISA kits to determine mineral, collagen type I, and BMP-2 contents. In addition, MG63 and SAOS-2 cells were seeded on tooth-derived materials and Bio-Oss^®^, and a comparison of cell responses in terms of adhesion and proliferation was carried out.	The demineralization process determined an increase in BMP-2 bioavailability, favouring the development of more effective, osteoinductive tooth-derived materials for bone regeneration and replacement.
Li et al. (2018) [69]	A prospective clinical study	Evaluation of DDM and Bio-Oss as bone substitutes in GBR for immediate placement of implants in periodontal post-extraction sites.	For 40 patients, DDM (size 0.5–1.0 mm) and Bio-Oss were used as bone grafts in the test group and control group, respectively. The implant stability, evaluated by Osstell Mentor, and marginal bone resorption, evaluated with x-ray examinations, were measured at T0, at 6 and 18 months after surgery.	The values of implant stability and marginal bone resorption were superimposable in the two groups.
Minetti et al. (2019) [38]	Multicenter Clinical Study	Studied the use of extracted tooth as autologous tooth graft after endodontic root canal therapies used for socket preservation and evaluated the implant insertion in regenerated bone with six-month follow up.	A total of 98 patients (29 men and 69 women) with an average age 53.7 years. Autologous tooth as a graft after the TT^®^ device procedure.	The success rate of the tooth graft procedure was 99.1% (one site was infected and lost the regeneration and the implant). In all cases, after all implants were inserted, complete osseointegration after proper healing period was achieved. After the healing period, hard and soft tissues were stable. The healing of soft tissues after grafting procedures was free of complications. The implant success rate was 98.94% (one implant failed).
Cardaropoli et al. (2019) [70]	Case report	To evaluate the regenerative potential of particles obtained from a crushed extracted tooth.	After tooth removal, the clean root was ground, and the dentin and cementum granules were grafted into a fresh extraction socket for a ridge preservation procedure.	Tissue healing was evaluated by histologic and radiologic analysis. The volume of the ridge was preserved. Histologically, a dentin–bone complex was reported. New bone formation was evident, with an intimate contact between bone and both dentin/cementum.
Kadkhodazadeh et al. (2020) [71]	In vitro study	To evaluate the osteopromoting ability of human tooth powder and compare it with a bovine xenograft, a synthetic material, and a demineralized freeze-dried bone allograft (DFDBA).	A total of 30 teeth were collected. The samples were ground to a powder with particles <500 µm. Osteoblast-like cells of MG-63 were cultured with the tooth powder, Cerabone, DFDBA, and Osteon II. Cell proliferation was assessed by the MTT assay at 24 and 72 h intervals.	Tooth powder was able to increase osteogenic cell proliferation in comparison with the bovine xenograft, the synthetic graft, and the DFDBA. However, its osteopromoting ability was less than the osteogenic materials.
Minetti et al. (2020) [37]	Multicenter pilot study	Evaluation of post-extraction site preservation with DDM from vital or endodontically treated teeth and a collagen membrane.	A total of 28 patients and 32 extractions. After 4 months, 32 biopsies were performed, and histological and histomorphometric analyses were evaluated.	There was no significant difference in the bone regeneration of sites where DDM was obtained from vital or endodontically treated teeth.
Minetti et al. (2020) [72]	Case reports	Present a case of alveolar socket preservation by using tooth graft material and one implant-supported rehabilitation	One 26-year-old women, nonsmoker, ASA-1 Autologous Deciduous Tooth-Derived Material.	Good results in terms of clinical and radiographic outcomes showing the absence of bone resorption process and the stability of soft tissues after two years.
Sánchez- Labrador et al. (2020) [73]	A split-mouth clinical trial	Evaluation of post-extraction site preservation of the included third molar with and without DDM.	A total of 15 patients, 30 lower third molars were extracted, 15 sites post-extraction were grafted with DDM (300–1200 μm) and 15 contralateral sites were left to heal without graft. At three and six months, the sites were evaluated with x-ray and probing of periodontal tissue.	Bone regeneration and reduced periodontal defect on the distal side of the lower second molar were found in the sites grafted with DDM.
Korsch et al. (2021) [74]	Retrospective study	Using autogenous dentin for lateral ridge augmentation.	For the tooth-shell method (TST): 28 patients (15 females, 13 males) with 34 areas and 38 implants, autogenous dentin slices were taken from teeth and utilized to restore lateral ridge deficiencies. The control was the bone-shell technique (BST), which was performed on the autogenous bone on 31 patients (16 females and 15 males) with 32 areas and 41 implants. In both situations, implants were put in at the same time. A follow-up three months following implantation.	Between the two groups, there were no appreciable variations in the overall number of problems. One implant with TST and one with BST both showed horizontal hard tissue loss of 1 mm and 0.5 mm, respectively.
Cervera-Maillo et al. (2021) [75]	Prospective clinical trial	To evaluate the efficacy of extracted teeth processed into bacteria-free particulate dentin in a Smart dentin grinder and then grafted immediately into alveolus post extraction or into bone deficiencies.	Ten healthy, partially edentulous patients with few teeth in the mandible were recruited in the study. After their own teeth were grinded, particulate teeth were placed in empty sockets and bone defects after teeth extractions. Furthermore, after 3,6, 12, and 24 months, core samples using a 3 mm trephine were obtained.	Particulate dentin grafts should be considered as an alternative material for sockets’ preservation, split technique, and sinus lifting. Clinically and histologically, the performance of the dentin graft is at least comparable to extensively used xenogeneic or allogenic biomaterials.
Minetti et al. (2022) [76]	Histological specimen study	The aim of the study was to explore the histomorphometric outcomes of tooth derivative materials used as bone substitute material in socket preservation procedure.	The use of a demineralised tooth as grafting material was evaluated for the preservation of the alveolus.	No significant difference was noted between the maxillary and mandibular sites, including defect type and section position.
Okubo et al. (2022) [77]	Case report	To evaluate the effectiveness of DDM for GBR in a patient with severe bone defect on the anterior upper jaw.	Ridge augmentation with DDM in a patient with atrophic maxilla and implant placement. After one year from the surgery a biopsy was executed.	Histological findings revealed the direct formation of new bone on DDM residue. Radiographs showed in the region of upper lateral incisor an increase in horizontal breadth of 3,16 mm after implant placement. The upper level’s breadth grew from 3.38 mm to 5.92 mm.
Pohl et al. (2022) [78]	Case report	Assess concerns about the biological response of these ATDGs in preparation for implant placement and subsequent osseointegration.	After 12 weeks of extraction socket healing, an implant with an acid-etched surface was placed using osseodensification osteotomy preparation and was retrieved after 16 weeks of integration. Histologic analysis revealed ≥ 64% of direct bone-to-implant contact at multiple regions of interest along the implant surface. Residual dentin particles have been searched in contact with the implant.	Residual dentin particles were scarce and were never found in contact with the implant. The autologous tooth-derived grafts did not interfere with implant osseointegration.
Minetti et al. (2022) [79]	Clinical trial	Evaluation of alveolar ridge augmentation with and without collagen membrane associated with DDM.	Six patients with defects requiring bone augmentation and DDM (406 µ m and 815 µ m with peaks up to 1110 µ m). In Group 1, DDM was associated with a resorbable membrane, and in group 2 only DDM was used. At four months, a bone tissue biopsy was performed.	Histological analysis showed more bone volume and vital bone in the sites where membrane had been used in association with DDB.

## Data Availability

Not applicable.

## Abbreviations

ALP	Alkaline Phosphatase
AR	Androgen Receptor
BMPs	Bone Morphogenetic Proteins
BSP	Bone Sialoprotein
DBM	Demineralized Bone Matrix
DDM	Demineralized Dentin Matrix
DFDBA	Demineralized Freeze-Dried Bone Allograft
DMP1	Dentin Matrix Protein 1
DSPP	Dentin Sialophosphoprotein
ER	Estrogen Receptor
FDA	Food and Drug Administration
FGFs	Fibroblast Growth Factors
GBR	Guided Bone Regeneration
GF	Germ-Free
HA	Hydroxyapatite
IGFII	Insulin Growth Factor II
LPS	Lipopolysaccharide
MMP	Matrix Metalloproteinases
MSC	Mesenchymal Stem Cell
NCP	Non-Collagen Proteins
NF-kB	Nuclear Factor kappa B
NFATc1	Nuclear Factor of Activated T Cells 1
OPG	Osteoprotegerin
OPN	Osteopontin
PDGF	Platelet-Derived Growth Factor
PRGF	Platelet Rich in Growth Factor
rhBMP-2	Recombinant Human BMP-2
RSV	Resveratrol
RUNX2	Runt-Related Transcription Factor 2
SCFA	Short-Chain Fatty Acids
SEM	Scanning Electron Microscope
TCP	Tricalcium Beta-Phosphate
TGF	Transforming Growth Factor
TRAF6	TNF Receptor Associated Factor 6
TT^®^	Tooth Transformer^®^
VEGF	Vascular Endothelial Growth Factor
